# P92 Benefits of a specialist multidisciplinary team approach to the management of *Aspergillus*-related chronic lung disease

**DOI:** 10.1093/jacamr/dlaf230.099

**Published:** 2025-12-04

**Authors:** Reece Weaver, Aarash Saleh, Christabelle Chen, Cristina Suarez, Jonathan Lambourne, Heinke Kunst

**Affiliations:** Barts Health NHS Trust, London, UK; Barts Health NHS Trust, London, UK; Barts Health NHS Trust, London, UK; Barts Health NHS Trust, London, UK; Barts Health NHS Trust, London, UK; Barts Health NHS Trust, London, UK

## Abstract

**Background:**

In June 2024, we established a monthly cross-site multidisciplinary team (MDT) to guide management of chronic fungal lung disease, consisting of respiratory and infectious diseases/microbiology physicians and highly specialist respiratory pharmacists. Management of fungal infections is complicated, involving protracted courses of high-cost antifungals with complex interaction and side-effect profiles. The MDT aims to optimize the holistic management and to improve the outcomes of patients with fungal lung disease across Barts Health NHS Trust.

**Objectives:**

To describe the patient cohort and decision making impacts of the novel fungal lung disease MDT on patient care and antifungal stewardship, and to ascertain if MDT recommendations (regarding antifungal therapy initiation and optimization of comorbidities) align with the practice points in the British Thoracic Society (BTS) clinical statement on *Aspergillus-*related chronic lung disease.

**Methods:**

Case notes were audited for all patients discussed since the MDT was established. Data were collected regarding diagnosis, antifungal treatment recommendations (including initiation, modification or cessation) and non-medication recommendations, using the definitions described in the BTS statement.

**Results:**

In the 14 months since establishment of the MDT, 22 patients have been discussed. The diagnoses of these patients (made on the basis of clinical features, radiological findings and mycological studies) include 15 with chronic pulmonary aspergillosis (CPA), 3 with allergic bronchopulmonary aspergillosis (ABPA) and 4 with ABPA/CPA overlap. Ten patients (45%) are currently on long-term triazole therapy (8 CPA, 1 ABPA, 1 overlap); all met the criteria in the BTS statement for initiating treatment for their respective conditions. Indications for treatment in patients with CPA and ABPA/CPA overlap were progressive radiological changes (*n*=8) and major haemoptysis (*n*=1). In the ABPA patient, the indication was two steroid-requiring exacerbations within 6 months. In three cases (14%), the MDT advised switching antifungal therapy due to toxicity attributed to the initial agent. Cessation of antifungal therapy was recommended for a further 6 patients (27%); the MDT monitored their progress as part of a watchful waiting approach. After discussion, 17 patients (77%) underwent optimization of their respiratory comorbidities (including inhaler technique review, prescription of mucolytic agents and referral to respiratory physiotherapy). All relevant patients were referred to smoking cessation services. 13 patients (59%) had documented recommendations related to optimization of their non-respiratory comorbidities (including diabetes therapy and nutritional status).

**Conclusions:**

This audit shows therapy is initiated and comorbidities are optimized largely in line with the BTS statement. Regular MDT discussion enabled discontinuation of long-term triazole therapy in six suitable cases, thereby reducing treatment costs and limiting the potential for development of antifungal resistance. When adverse effects occur, newer (higher-cost and broader-spectrum) triazoles are often indicated as an alternative, due to their lower incidence of toxicity. Specialist pharmacist involvement before their recommendation promotes stewardship of newer triazoles but ensures their use when necessary. MDT discussion also prompts optimization of comorbidities, improving overall wellbeing and avoiding costly hospital admission. In particular, early optimization of respiratory comorbidities may prevent exacerbations, which often require additional antimicrobial therapy, with associated cost and risk of antimicrobial resistance.

